# Pre- and post-traumatic boric acid therapy prevents oxidative stress-mediated neuronal apoptosis in spinal cord injury

**DOI:** 10.22038/ijbms.2024.81531.17649

**Published:** 2025

**Authors:** Turan Kandemir, Ibrahim Sogut, Zeki Serdar Ataizi, Betul Can, Aysegul Oglakci-Ilhan, Dilek Burukoglu-Donmez, Gungor Kanbak

**Affiliations:** 1 Department of Neurosurgery, Eskisehir Osmangazi University Faculty of Medicine, Eskisehir, Turkey; 2 Department of Biochemistry, Demiroglu Bilim University, Medical Faculty, Istanbul, Turkey; 3 Department of Neurosurgery, Eskişehir Yunus Emre State Hospital, Eskisehir, Turkey; 4 Department of Medical Biochemistry, Eskisehir Osmangazi University Faculty of Medicine, Eskisehir, Turkey; 5 Department of Medical Laboratory Techniques, Eldivan Health Services Vocational School, Cankırı Karatekin University, Cankırı, Turkey; 6 Department of Histology and Embryology, Eskisehir Osmangazi University Faculty of Medicine, Eskisehir, Turkey

**Keywords:** Apoptosis, Boric acid, Neuroprotective, Oxidative stress, Spinal cord injury

## Abstract

**Objective(s):**

In our study, the neuroprotective efficacy of pre- and post-traumatic applications of boric acid (BA) in rats with experimentally induced spinal cord injury (SCI) was investigated.

**Materials and Methods:**

The experimental animals were divided into four groups: control group (C), SCI group (SCI), BA-treated group before SCI (BA+SCI), and BA-treated group after SCI (SCI+BA). Forty-eight hours after SCI, biochemical levels of malondialdehyde (MDA), total oxidant status (TOS), total antioxidant status (TAS), oxidative stress index (OSI), and cytochrome c (Cytc) and caspase-3 (Casp3) expressions were measured in the spinal cord tissues and were examined histologically.

**Results:**

After SCI, oxidative stress markers, such as MDA, TOS, and OSI, and apoptosis markers Cytc and Casp3 showed an increase in levels compared to Group C. The oxidative stress markers that increased after SCI decreased with BA+SCI application, while Cytc level, one of the apoptosis markers that increased after SCI, decreased in both groups with BA application. Cell, myelin, ependymal damage, and hemorrhage levels increased after SCI compared to Group C. These histological markers increased after SCI and decreased after BA+SCI. BA was found to reduce SCI-induced oxidative stress and oxidative stress-induced apoptosis.

**Conclusion:**

BA administered before SCI was shown to be more effective in protecting neural damage.

## Introduction

Spinal cord injury (SCI) is one of the most important health problems of today as it is a serious neurological problem that affects modern society physically, psychosocially, and economically. In many studies, traffic accidents are the first cause of SCI (1, 2). Other causes include work accidents, falls from height, gunshot wounds, and sports injuries. Although the worldwide prevalence of SCI is unknown, a meta-analysis found that the incidence of traumatic SCI was 26.48 per million people (3). Understanding the pathophysiology of SCI is important in designing a therapeutic approach. The pathophysiological process of SCI consists of two stages: primary injury and secondary injury. The mechanical damage that initially occurs in SCI is referred to as primary damage and consists of acute, physiologic, and structural disruption of axons (4). Although the primary damage is limited to the injury site, it triggers the onset of many subsequent physiopathological processes, leading to secondary damage. Secondary damage can last for months. During this period, the blood-spinal cord barrier is disrupted, and apoptosis, increased peripheral inflammatory cells, reactive oxygen/nitrogen species (ROS/RNS) generation, intracellular calcium accumulation, activation of proteases and caspases, lipid peroxidation, and glutamate excitotoxicity such as compression, tearing, shearing, and stretching are observed (5-7).

As mentioned above, overproduction of ROS exacerbates oxidative stress, inflammatory response, and various cell death pathways, which play a crucial role in the secondary injury process (8, 9). Enzymatic and nonenzymatic antioxidant mechanisms inactivate free radicals formed under physiological conditions. Enzymatic antioxidants (e.g., catalase (CAT), superoxide dismutase (SOD), glutathione peroxidase (GPx), etc.) cannot respond to the excessive increase of free radicals in the tissue after trauma to the same degree. There is a need to increase the concentration of nonenzymatic antioxidants (e.g., vitamin C, vitamin E, glutathione) in the body that act as radical scavenging agents (10). Under physiological conditions, mitochondria are the largest source of cellular ROS (e.g., superoxide, hydroxyl radicals, hydrogen peroxide, lipid peroxide, and peroxynitrite) as they utilize oxygen by cells during oxidative phosphorylation. However, SCI causes mitochondrial dysfunction, further increasing ROS production (11). Furthermore, ROS uptake by microglia and leukocytes is further increased after SCI (12). Under normal conditions, ROS are balanced by various biochemical processes, such as antioxidant systems. However, after SCI, increased ROS formation and also this imbalance between free radicals and antioxidants leads to the formation of oxidative stress, especially in lipids, proteins, and nucleic acids (13-15). In addition, the blood-brain barrier is disrupted due to endothelial damage caused by free oxygen radicals. As a result, there is an accumulation of ROS at the site of injury. Free radical damage occurs more frequently due to the low superoxide dismutase (SOD), catalase, and glutathione peroxidase (GPx) activities in this region (16). Apoptosis is responsible for cell death after traumatic SCI. Apoptotic processes are triggered by the action of reactive oxygen species (ROS). The increased amount of ROS increases mitochondrial membrane permeability. It leads to the release of pro-apoptotic molecules, such as cytochrome c, from mitochondria and an increase in the level of Caspase-3 from the cysteine protease family, causing apoptosis (17-19). The mechanism of apoptosis in spinal cord injuries depends on caspase-3 and calpain. Following injury, apoptosis mediated by caspase, cytochrome-c release into the cytosol, and an increase in the bax/bcl-2 ratio are observed in spinal cord lesions. Thus, apoptosis is observed in neurons and glial cells (20).

Recent studies have revealed that one of the main functions of mitochondria is to regulate apoptosis. Neurons are very rich in mitochondria and are the cell type with the highest energy consumption; hence, they have a higher demand for mitochondrial ATP production. Mitochondria are the main source of ROS/RNS production and, therefore, a potential therapeutic target (21). Studies have shown free radical-mediated mitochondrial dysfunction as a significant contributor to secondary damage following SCI. Oxidative damage and mitochondrial dysfunction are interrelated processes that also catalyze other secondary injury events (22).

The most effective therapeutic intervention for SCI is the prevention of secondary injuries, especially by using various antioxidants to reduce the reduction of oxygen free radicals. (13, 16). Boric acid (BA), which is used in this study, is an important trace element for organisms. BA shows Lewis acid properties, forms complexes with glycoproteins and glycolipids containing hydroxyl groups, and has Ca+ chelator properties. It also has antioxidant, antiapoptotic, and anticancer properties as well as oxidative stress-reducing properties (13, 14, 18, 19). Previously, our research team demonstrated the oxidative stress-reducing effect of BA in rats with traumatic brain injury (13). In this study, we investigated the protective role of pre and after-boric acid administration in oxidative stress-mediated mitochondrial dysfunction and neuronal apoptosis.

## Materials and Methods

In the current experiment, 56 male mature Sprague Dawley rats weighing 250-260 g were used. The rats were kept in standard animal care cages, with a 12-hour day/night cycle, at a constant temperature of 22 ^°^C±30 ^°^C and a constant humidity (55%±5%). All experimental procedures were conducted in accordance with the guidelines of Eskisehir Osmangazi University Animal Ethics Committee (Protocol no: 847-1/2022) and with the Guide for the Care and Use of Laboratory Animals prepared by the National Academy of Sciences.

**Figure 1:**
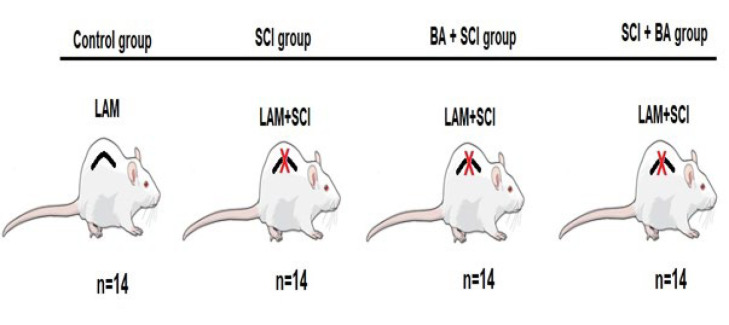
Experimental groups, laminectomy (LAM), spinal cord injury (SCI), 100 mg/kg boric acid (BA)

The rats were randomly divided into four groups. Control group (C; n=14): Laminectomy without any injury to the spinal cord was performed. SCI (n=14): Laminectomy and SCI was performed. BA+SCI group (n=14): Intraperitoneal (IP) BA was administered (100 mg/kg)(13, 14) one hour before laminectomy and induction of SCI. SCI+BA group (n=14): BA was administered intraperitoneally (100 mg/kg) one hour after laminectomy and inducing SCI (Figure 1).

Due to limited access to spinal cord tissue in experimental SCI, the number of groups was formed as n=14. Seven experimental animals were used for biochemical studies in each group, and the other seven were used for histological and molecular studies.

### Preparation and implementation of BA

BA solution was freshly prepared in 0.5 ml saline solution (SS) containing 100 mg/kg doses of BA (Merck, Darmstadt, Germany) just before the study. According to the groups’ content, BA was administered via the IP route one hour before SCI and one hour after SCI(23-26).

### Traumatic SCI model and procedure

The experimental animals were subjected to the clip compression model described by Rivlin and Tator to induce spinal trauma (27). During the surgical procedure, a Yasargil FE 720K (with a closing force of 110 g, Aesculap/Germany) clip was used. The surgical procedure was performed on the animals under general anesthesia with intraperitoneal xylazine hydrochloride (10 mg/kg) and ketamine hydrochloride (90-100 mg/kg). After anesthesia, the rats were placed on the operation table in the prone position, the dorsal region was shaved, and local antisepsis was applied with polyvinyl iodine. A single-level total laminectomy was performed by placing the laminae between dorsal 7-10 vertebrae through a midline surgical incision. After careful placement without damaging the dura, the aneurysm was cord-clipped with an aneurysm clip. After one minute, the clip was removed, and the surgical procedure was completed. Muscles and skin were closed in layers with 3-0 silk sutures after all bleeding was stopped. It was accepted that SCI was successfully established as the hindlimbs were paralyzed, and tail spasm was observed after the surgical procedure. After the operation, the rats were placed in their cages, and an antiseptic solution was used daily to prevent wound infection. The rats’ bladders were emptied manually twice a day. 

### Collection and evaluation of samples

**Figure 2:**
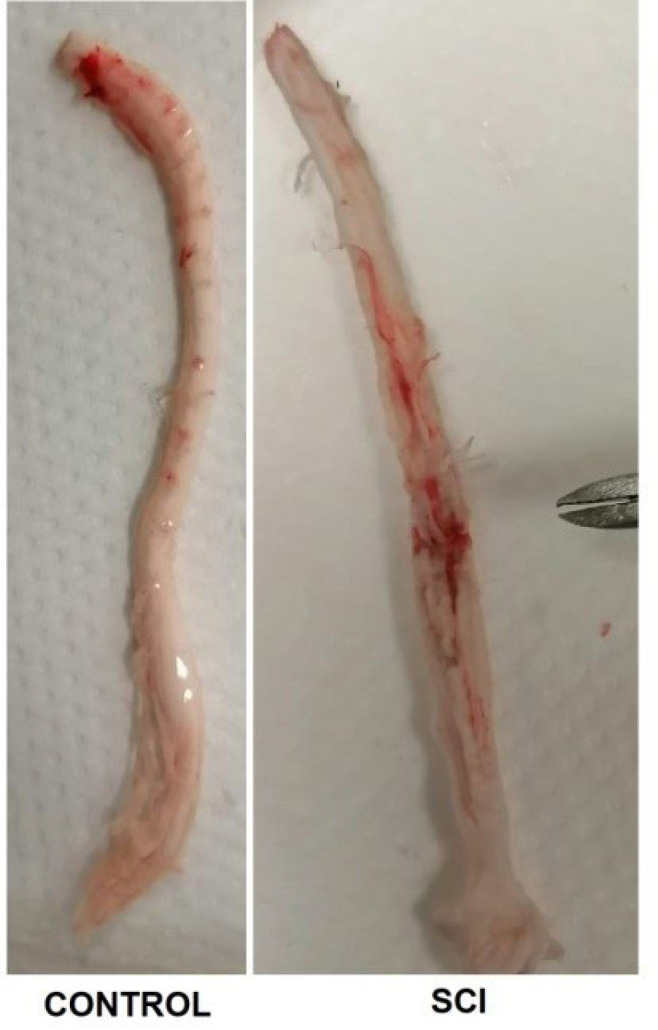
Spinal cords of control and spinal cord injury (SCI) groups - The damaged area is at the level of the scissors

Forty-eight hours after the surgical procedure, the rats were sacrificed under general anesthesia, blood was drawn from their heart directly, and then their spinal cords were excised due to total laminectomy (28)(Figure 2). The obtained tissue pieces were washed in SS and immediately stored at -80 ^°^C for biochemical studies. The tissue pieces were then placed in RNAlater solution (GeneMark, Maharashtra, India) and stored at -20 ^°^C for molecular studies. For histological examination, the samples were placed in 10% neutral formalin fixative and fixed for 48 hr**.**

### Biochemical measurements

Total antioxidant status (TAS; mmol Trolox eq/l), total oxidant status (TOS; µmol H2O2 eq/l), and oxidative stress index (OSI; arbitrary unit) were detected using commercial kits (Rel Assay Diagnostics; Gaziantep, Turkey). The OSI levels in the sample were determined as the ratio of the TOS level to the TAS level. Lipid peroxidation was quantified at 532 nm by the measurement of malondialdehyde (MDA) reacted with the thiobarbituric acid according to the method of Ohkawa *et al*. The results were expressed in nmol/mg protein(29). The tissue protein amounts were measured by a BCA protein assay kit (iNtRON Biotechnology, Gyeonggi-do, Korea). 

### Molecular measurements

Total RNAs were isolated using the RNAzol RT solution (MRC, Cincinnati, OH, USA) according to the manufacturer’s instructions for quantifying mRNA expression in spinal cords. After completion of RNA isolation, the RNA concentration and purity were calculated with NanoDrop 2000 (Thermo Scientific, MA, USA). For this purpose, 1 µl RNA samples were pipetted in the device for determination of 260/280 and 260/230 ratios. Concentrations of all RNA samples were equalized before reverse transcription. RNAs were reverse transcribed into cDNA by using Script cDNA Synthesis Kit (Jena Bioscience, Germany). The resulting cDNA was amplified by qRT-PCR by using qPCR EvaGreenMaster (Solis BioDyne, Estonia). The real-time conditions were carried out on the CFX-96 RT-PCR System (Bio-Rad, CA, USA) as follows: 50 ^°^C for 2 min, 95 ^°^C for 2 min, and then 35 cycles of 95 ^°^C for 15 sec; 55 ^°^C for 20 sec, and 72 ^°^C for 20 sec. The relative mRNA transcript levels were calculated according to the 2–∆∆Ct method and the relative expression of each gene was normalized to that of glyceraldehyde-3-phosphate dehydrogenase (GAPDH). Primers were obtained from LGC Biosearch Technologies (Novato, CA, USA)(Table 1). All measurements were obtained in triplicates, and the specificity of amplicons was verified by melting curve analysis. The specific primers used are shown in Table 1.

### Histological examination

The fixed specimens were subjected to routine histological follow-up methods and embedded in paraffin blocks. Tissue sections of 5 mm thickness were taken from the paraffin blocks with a microtome. The sections were stained with hematoxylin and eosin for microscopic examination. The samples obtained from all groups were evaluated at light microscopic level with an Olympus BH-2 (Olympus Corp., Tokyo, Japan) microscope, and all preparations were photographed with an Olympus DP-70 (Olympus Corp., Tokyo, Japan) digital camera. All specimens were evaluated and scored for cellular damage/ischemia, myelin damage/vacuolization, ependymal cell damage, and hemorrhage (30, 31).

### Statistical analysis

SPSS software, version 22.0 for Windows (SPSS, Inc., Chicago, Illinois), was used for the statistical analysis of biochemical and histological data. One-way ANOVA was applied to the data that followed normal distribution, while the Kruskal-Wallis test was applied to the data that did not follow normal distribution. The results were presented as mean±standard deviation and median (minimum-maximum); *P*<0.05 was considered statistically significant.

## Results

### Biochemical, molecular, and histological findings

When the MDA levels were compared between the groups, as shown in Table 2, the MDA level increased after spinal injury. It decreased significantly in the group treated with BA before neural injury (BA+SCI)(*P*<0.01). The TOS level increased significantly after SCI compared to the control group (*P*<0.01). The TOS level in the BA+SCI group, the increase caused by SCI, was significantly reduced (*P*<0.05). When the TAS levels were compared between the groups, although antioxidant levels increased with both BA treatments, no significant difference was observed (*P*>0.05). The OSI level of the SCI group was significantly increased compared to the control group (*P*<0.001). In the BA+SCI group, the increase in OSI caused by SCI was significantly reduced (*P*<0.01)(Table 2).

Molecular apoptotic markers caspase-3 and cytochrome c levels were significantly increased after SCI compared to the control group (*P*<0.01 and *P*<0.001, respectively). The cytochrome c levels were significantly decreased in the BA-treated groups compared to the SCI group. This decrease was *P*<0.05 in the BA+SCI group and *P*<0.01 in the SCI+BA group (Table 2).

**Figure 3:**
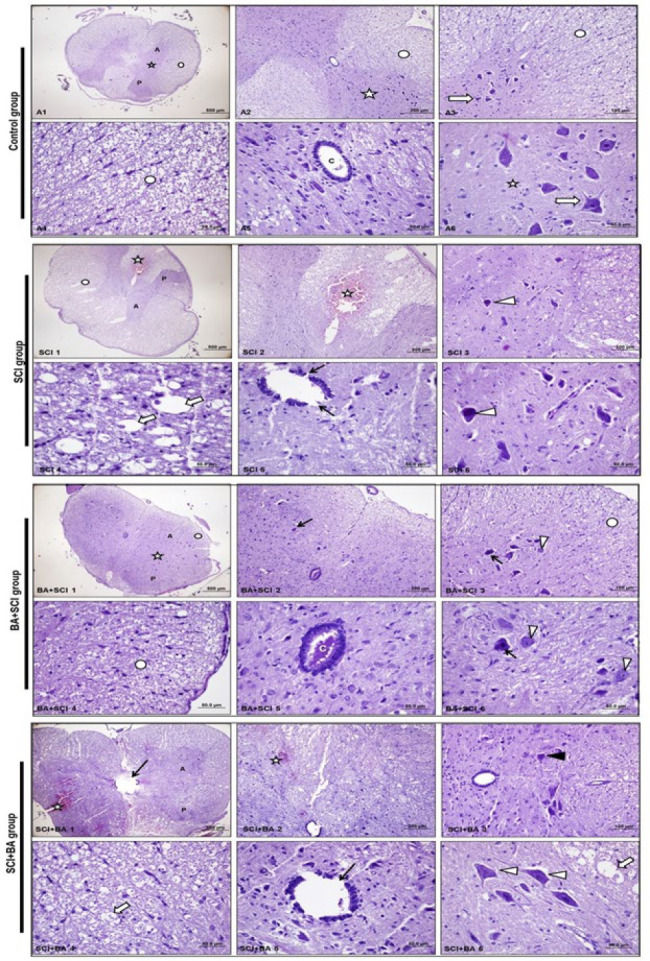
Histological H&E staining of spinal cord sections from all groups

The histopathological examination of the control group was normal (Figure 3, Table 2). In the examination of the SCI group, ischemia in the motor neurons in the anterior horn in the grey matter of the medulla spinal cord and damage in ependymal cells were observed. Intense degenerative changes and hemorrhagic areas were observed in myelin in the white matter (Figure 3, Table 2). The histopathologic examination of the BA+SCI group revealed reduced damage. Although several ischaemic cells were observed in the anterior horn in the grey matter, near-normal multipolar motor neurons with euchromatic nuclei and cell bodies were observed in general examination (Figure 3, Table 2). Although reduced damage was noted in the SCI+BA group, hemorrhagic areas in the white matter, degeneration of myelin, and partial damage to ependymal cells were observed. Although a few ischaemic cells were observed in the anterior horn, overall examination revealed near-normal multipolar motor neurons with euchromatic nuclei and cell bodies (Figure 3, Table 2).

## Discussion

Despite all technological and scientific advances, no definitive medical or surgical technique has yet been developed to ensure complete neural improvement in SCI. To reduce the effects of the damage, ensuring adequate spinal cord perfusion in intensive care, using neuroprotective agents such as methylprednisolone sodium succinate, decompressive surgery, and many neuroprotective and neuroregenerative therapy methods have been investigated by creating experimental SCI (23, 27, 32, 33). 

With our study, the effects of BA on oxidative stress and apoptosis before and after SCI were investigated for the first time. BA is a trace element for living things. BA plays an important role in cell membrane functions and enzyme reactions, such as affecting the transduction of signals or regulatory ions across cell membranes (34). In the literature, the studies conducted with BA have increased recently, and its effects on nerve cells have also been investigated. In a study, it was reported that boron deprivation caused symptoms such as decreased electrical activity of the brain, loss of consciousness and psycho-motor activity, decreased movement and skills, and poor short-term memory (35). In the literature, there are studies showing that BA is protective against oxidative stress and has neuroprotective properties (13, 14, 36, 37). MDA levels, one of the oxidative stress markers, show an increase during the secondary injury (32, 38). In our study, the MDA level, which is an indicator of the peroxidation of polyunsaturated fatty acids in cell membranes, increased significantly. In addition, the TOS and OSI levels, which are other oxidative stress indicators, increased with SCI. Previous studies investigating the protective effect of minocycline and genistein in rats with SCI also showed an increase in TOS levels after SCI (38, 39). TAS levels have previously been shown to decrease after SCI (40). In our study, the TAS levels decreased moderately after SCI.

The exact mechanism of antioxidant and neuroprotective effects of BA has not yet been established. The effect of BA in the body varies depending on the prescribed dose. In a study, it was shown that low doses of BA showed neuroprotective properties in neurotoxicity induced by aluminum chloride in mouse brains, while high doses did not prevent neurotoxicity (41). In another experimental study, it was shown that 100 mg/kg BA had a positive effect on the recovery of axon and myelin damage in sciatic nerve injury (37). Since we have seen the protective effect of 100/kg BA dose in our other studies, we have previously conducted on brain cells (13, 14). We applied the same concentration of BA in this study. Biochemical markers (MDA, TOS, and OSI levels), which increased as a result of SCI injury, were significantly decreased with BA administration before trauma in this study. A significant decrease in the MDA value was achieved with BA administered after previously induced traumatic brain injury (13). In another study, it was shown that *in vitro* ethanol decreased the oxidative stress level on rat brain synaptosomes under *in vitro* conditions by applying BA (14). Again, the increased MDA level in rat brain synaptosomes by sodium fluoride induction was reduced by applying BA (36). BA has been previously reported to exert its antioxidant effect by inhibiting lipid peroxidase and maintaining the integrity of the cell membrane (1, 36, 42). However, there are studies in which nonsignificant antioxidant increases were observed with BA administration, as well as studies in which no increase was observed (14, 43). In our study, the TAS levels decreased after SCI, but administration of BA before and after injury caused moderate increases. In our study, a more effective decrease in the MDA, TOS, and OSI levels was found with BA administered before SCI. This situation emphasized the protective effect of BA on cell membrane integrity rather than its therapeutic properties.

After traumatic SCI, apoptotic cell death markers, such as cytochrome c, caspase-3, caspase-9, and Apaf-1 (apoptotic peptidase activating factor 1), which are markers of apoptosis, increased with the decrease in the activity of mitochondrial respiratory enzymes (44-46). In our study, parallel with previous studies, cytochrome c and caspase-3 levels were found to increase after SCI. In our study, it was observed that BA decreased the cytochrome c level, which increased after cord injury, and decreased caspase-3 activities moderately in the groups administered before and after SCI. It was previously found that oxidative stress in liver cells increased as a result of chronic alcohol use. As a result, the caspase-3 activity and DNA fragmentation, which are among the markers of apoptosis, increased. BA administration with alcohol caused a decrease in oxidative stress and thus decreased caspase-3 and DNA fragmentation, which are markers of apoptosis (18). In our study, increased oxidative stress after SCI caused apoptosis to be triggered, and we suggest that BA applications decreased the amount of Ca+ and, indirectly, cytochrome c levels during cell death by protecting cell membrane integrity.

In addition, histological features of the spinal cortex of rats with acute traumatic injury in previous studies showed that hematoma, edema, and cell damage increased after SCI (47, 48). In addition, it was found that NeuN and Nissl staining levels, which are markers of mature neurons, decreased after SCI, and the number of TUNEL-positive cells, which is an indicator of apoptosis, increased after SCI (49). In our study, in parallel with the previous studies, it was found that cell, myelin, and ependymal cell damage, as well as hemorrhage levels, increased after SCI. In a study in which BA was applied after spinal cortex ischemia, edema, hemorrhage, and neuronal degeneration were found to be decreased (48). In a study conducted by our group, increased edema and necrotic neuron numbers after traumatic brain injury decreased after BA application (13). In the histological examination of our study, increased cell, myelin, ependymal damage, and hemorrhage areas after SCI showed significant changes in the groups in which BA was applied before and after SCI. This healing was found to be more effective in the group administered BA before trauma. Histologic results were consistent with our biochemical results.

**Table 1:** Primers used in real-time polymerase chain reaction analysis for Caspase-3 and Cytochrome-c

Primer Name	Forward Primer Sequence (5′-3′)	Reverse Primer Sequence (3′-5′)
Caspase-3 (NM_009810.3)	TGACTGGAAAGCCGAAACTC	GTAGAGTAAGCATACAGGAAGTCAG
Cytochrome-c (NM_007808.5)	AAGGGAGGCAAGCATAAGAC	ATTCTCCAAATACTCCATCAGGG
GAPDH (Housekeeping) (NM_008084.3)	CCTCGTCCCGTAGACAAAATG	TGTAGTTGAGGTCAATGAAGGG

**Table 2:** Comparison of all groups with biochemical, molecular and histological findings

Groups → Biochemical Parameters ↓	Control	SCI	BA+SCI	SCI+BA	p
MDA (nmol/mg protein)	13.94±3.23	17.69±2.6	11.25±3.55	15.40±3.02	SCI & BA+SCI **
TOS (µmol H_2_O_2_ eq/l	41.50±8.4	62±11.16	48.75±10.69	54.5±7.3	Control & SCI ** SCI & BA+SCI *
TAS (mmol Trolox eq/l)	2.86±1.17	2.06±0.49	3.23±1.4	2.34±0.28	No significant
OSI (OSI; arbitrary unit)	16.17±7.62	31.25±8.21	17.36±6.76	23.59±4.39	Control & SCI *** SCI & BA+SCI **
Groups → Molecular Parameters ↓	Control	SCI	BA+SCI	SCI+BA	p
Cytochrome c	1.0	2.183±0.58	1.461±0.36	1.233±0.61	Control & SCI *** SCI & BA+SCI * SCI & SCI+BA **
Caspase 3	1.0 (1-1)	2.02 (1.77-2.17)	1.8 (1.23-2.25)	1.42 (1.06-1.71)	Control & SCI **
Groups→ Histological Parameters ↓	Control	SCI	BA+SCI	SCI+BA	p
Cell damage/Ischemia	0 (0-0)	3 (2-3)	1 (1-2)	2 (1-2)	Control & SCI **** Control & SCI+BA * BA+SCI & SCI *
Myelin damage/Vacuolization	0 (0-0)	3 (2-3)	0 (0-1)	2 (1-2)	Control & SCI **** Control & SCI+BA * BA+SCI & SCI ***
Ependymal cell damaged	0 (0-0)	2 (2-3)	0 (0-1)	1 (1-2)	Control & SCI **** Control & SCI+BA * BA+SCI & SCI ***
Hemorrhage	0 (0-0)	3 (2-3)	0 (0-1)	0 (0-1)	Control & SCI **** BA+SCI & SCI *** SCI+BA & SCI *

Spinal cord injury (SCI), boric acid+spinal cord injury (BA+SCI), spinal cord injury+boric acid (SCI+BA), malondialdehyde (MDA), total oxidant status (TOS), total antioxidant status (TAS), oxidative stress index (OSI), * *P<*0.05, ** *P<*0.01, ***P<0.001

Control group: In the light microscopic examination, the canalis centralis (c), anterior horn (A), and posterior horn (P) structures of the medulla spinalis and grey matter (), multipolar motor neurons in the anterior horn with their euchromatic nuclei and cell bodies (), and axons and myelin sheaths in the white matter () are seen in normal structure (A1-A6) at different magnifications (bar: 500 µm, bar: 200 µm, bar: 100 µm, bar: 50.0 µm, HE). SCI group: The light microscopic examination shows ischemia () in motor neurons in the anterior horn in the grey matter of the medulla spinalis (A) and damage () in ependymal cells at different magnifications. In addition, intense degenerative changes in myelin () and hemorrhagic areas () in the white matter () are remarkable (B1-B6), (bar: 500 µm, bar: 200 µm, bar: 100 µm, bar: 50.0 µm, HE). BA+SCI group: The light microscopic examination of the medulla spinalis reveals reduced damage. The grey matter with anterior horn (A) and posterior horn (P) structures (), white matter with axons and myelin sheaths (), and canaliculus centralis structure (C), and ependymal cells lining the canaliculus centralis are seen in a near-normal histological structure. In the grey matter, a few ischaemic cells (→) are observed in the anterior horn. However, in general examination, near-normal multipolar motor neurons () with euchromatic nuclei and cell bodies are observed (C1-C6), (bar: 200 µm, bar: 100 µm, bar: 50.0 µm, bar: 20.0 µm, HE). SCI+BA group: The light microscopic examination of the medulla spinalis revealed reduced damage, hemorrhagic areas in the white matter (), degeneration of myelin (), partial damage to ependymal cells (→), and a few ischaemic cells in the anterior horn (), but in general examination, near-normal multipolar motor neurons () with euchromatic nuclei and cell bodies were observed (D1-D6), (bar: 200 µm, bar: 100 µm, bar: 50.0 µm, bar: 20.0 µm, HE)

## Conclusion

Our data demonstrated that neuronal injury is elevated after SCI via oxidative stress. Moreover, neuronal apoptosis might be triggered by mitochondrial cytochrome c release and depends on elevated caspase-3 activities. We also showed that boric acid might be effective as an antioxidative agent in SCI, especially in boric acid pre-treatment. However, the antiapoptotic role of boric acid is limited.

## Declaration

We have not used any artificial intelligence tools or technologies to prepare this manuscript.

## References

[1] Smith E, Fitzpatrick P, Murtagh J, Lyons F, Morris S, Synnott K (2018). Epidemiology of traumatic spinal cord injury in Ireland, 2010-2015. Neuroepidemiology.

[2] Halvorsen A, Pettersen A, Nilsen S, Halle KK, Schaanning EE, Rekand T (2019). Epidemiology of traumatic spinal cord injury in Norway in 2012–2016: a registry-based cross-sectional study. Spinal Cord.

[3] Lu Y, Shang Z, Zhang W, Pang M, Hu X, Dai Y (2024). Global incidence and characteristics of spinal cord injury since 2000–2021: a systematic review and meta-analysis. BMC Med.

[4] Marcon RM, Barros Filho TEPd, Oliveira RP, Cristante AF, Taricco MA, Colares G (2010). Experimental study on the action of methylprednisolone on Wistar rats before spinal cord injury. Acta Ortopédica Brasileira.

[5] Silva NA, Sousa N, Reis RL, Salgado AJ (2014). From basics to clinical: A comprehensive review on spinal cord injury. Prog Neurobiol.

[6] Fatima G, Sharma V, Das S, Mahdi A (2015). Oxidative stress and antioxidative parameters in patients with spinal cord injury: Implications in the pathogenesis of disease. Spinal cord.

[7] Ma Y, Li P, Ju C, Zuo X, Li X, Ding T (2022). Photobiomodulation attenuates neurotoxic polarization of macrophages by inhibiting the notch1-HIF-1α/NF-κB signaling pathway in mice with spinal cord injury. Front Immunol.

[8] Chen M, Chen S, Lin D (2016). Carvedilol protects bone marrow stem cells against hydrogen peroxide-induced cell death via PI3K-AKT pathway. Biomed Pharmacother.

[9] Dimitrijevic MR, Danner SM, Mayr W (2015). Neurocontrol of movement in humans with spinal cord injury. Artif Organs.

[10] Shah AA, Gupta A (2020). Antioxidants in health and disease with their capability to defend pathogens that attack apple species of Kashmir. Plant Antioxid Health.

[11] Wingrave JM, Schaecher KE, Sribnick EA, Wilford GG, Ray SK, Hazen-Martin DJ (2003). Early induction of secondary injury factors causing activation of calpain and mitochondria-mediated neuronal apoptosis following spinal cord injury in rats. J Neurosci Res.

[12] D’Autréaux B, Toledano MB (2007). ROS as signalling molecules: Mechanisms that generate specificity in ROS homeostasis. Nat Rev Mol Cell Biol.

[13] Ataizi ZS, Ozkoc M, Kanbak G, Karimkhani H, Donmez DB, Ustunisik N (2021). Evaluation of the neuroprotective role of boric acid in preventing traumatic brain injury-mediated oxidative stress. Turk Neurosurg.

[14] Sogut I, Oglakci A, Kartkaya K, Ol KK, Sogut MS, Kanbak G (2015). Effect of boric acid on oxidative stress in rats with fetal alcohol syndrome. Exp Ther Med.

[15] Huang W, King V, Curran O, Dyall S, Ward R, Lal N (2007). A combination of intravenous and dietary docosahexaenoic acid significantly improves outcome after spinal cord injury. Brain.

[16] Yazihan N, Uzuner K, Salman B, Vural M, Koken T, Arslantas A (2008). Erythropoietin improves oxidative stress following spinal cord trauma in rats. Injury.

[17] Emery E, Aldana P, Bunge MB, Puckett W, Srinivasan A, Keane RW (1998). Apoptosis after traumatic human spinal cord injury. J Neurosurg.

[18] Sogut I, Paltun SO, Tuncdemir M, Ersoz M, Hurdag C (2018). The antioxidant and antiapoptotic effect of boric acid on hepatoxicity in chronic alcohol-fed rats. Can J Physiol Pharmacol.

[19] Hacioglu C, Kar F, Kacar S, Sahinturk V, Kanbak G (2020). High concentrations of boric acid trigger concentration-dependent oxidative stress, apoptotic pathways and morphological alterations in DU-145 human prostate cancer cell line. Biol Trace Elem Res.

[20] Friedlander RM (2003). Apoptosis and caspases in neurodegenerative diseases. N Engl J Med.

[21] Schwarz TL (2013). Mitochondrial trafficking in neurons. Cold Spring Harb Perspect Biol.

[22] Bains M, Hall ED (2012). Antioxidant therapies in traumatic brain and spinal cord injury. Biochim Biophys Acta.

[23] Resnick DK (2013). Topic Foreword. Neurosurgery.

[24] Center SL (2016). Spinal cord injury (SCI) 2016 facts and figures at a glance. J Spinal Cord Med.

[25] Hacıoğlu C, Kar F, Şentürk H, Kanbak G (2018). Effects of boric acid on electrolyte balance and lipid profile against renal ischemia reperfusion injury. Biol Divers Conserv.

[26] Kar F, Hacioglu C, Senturk H, Donmez DB, Kanbak G (2020). The role of oxidative stress, renal inflammation, and apoptosis in post ischemic reperfusion injury of kidney tissue: The protective effect of dose-dependent boric acid administration. Biol Trace Elem Res.

[27] Ross IB, Tator CH (1993). Spinal cord blood flow and evoked potential responses after treatment with nimodipine or methylprednisolone in spinal cord-injured rats. Neurosurgery.

[28] Anjum A, Yazid MDi, Fauzi Daud M, Idris J, Ng AMH, Selvi Naicker A (2020). Spinal cord injury: Pathophysiology, multimolecular interactions, and underlying recovery mechanisms. Int J Mol Sci.

[29] Ohkawa H, Ohishi N, Yagi K (1979). Assay for lipid peroxides in animal tissues by thiobarbituric acid reaction. Anal Biochem.

[30] Huntemer-Silveira A, Patil N, Brickner MA, Parr AM (2021). Strategies for oligodendrocyte and myelin repair in traumatic CNS injury. Front Cell Neurosci.

[31] Smith PM, Jeffery ND (2006). Histological and ultrastructural analysis of white matter damage after naturally-occurring spinal cord injury. Brain Pathol.

[32] Yu C, Gui F, Huang Q, Luo Y, Zeng Z, Li R (2022). Protective effects of muscone on traumatic spinal cord injury in rats. Ann Transl Med.

[33] Yao R, Ren L, Wang S, Zhang M, Yang K (2021). Euxanthone inhibits traumatic spinal cord injury via antioxidative stress and suppression of p38 and PI3K/Akt signaling pathway in a rat model. Transl Neurosci.

[34] Nielsen FH (2008). Is Boron nutritionally relevant?. Nutr Rev.

[35] Penland JG (1998). The importance of boron nutrition for brain and psychological function. Biol Trace Elem Res.

[36] Hacioğlu C, Fatih K, Senturk H, Kanbak G (2018). Neuroprotective effects of boric acid against fluoride toxicity on rat synaptosomes. Med Sci Discov.

[37] Kızılay Z, Erken HA, Çetin NK, Aktaş S, Abas Bİ, Yılmaz A (2016). Boric acid reduces axonal and myelin damage in experimental sciatic nerve injury. Neural Regen Res.

[38] Aras M, Altas M, Motor S, Dokuyucu R, Yilmaz A, Ozgiray E (2015). Protective effects of minocycline on experimental spinal cord injury in rats. Injury.

[39] Bal E, Hanalioğlu Ş, Apaydın AS, Bal C, Şenat A, Öcal BG (2021). Anti-inflammatory and antioxidative effects of genistein in a model of spinal cord injury in rats. Asian Biomed.

[40] Yu L, Qian J (2020). Dihydrotanshinone I alleviates spinal cord injury via suppressing inflammatory response, oxidative stress and apoptosis in rats. Med Sci Monit.

[41] Colak S, Geyikoğlu F, Keles ON, Türkez H, Topal A, Unal B (2011). The neuroprotective role of boric acid on aluminum chloride-induced neurotoxicity. Toxicol Ind Health.

[42] Cikler-Dulger E, Sogut I (2020). Investigation of the protective effects of boric acid on ethanol induced kidney injury. Biotech Histochem.

[43] Balci Yuce H, Toker H, Goze F (2014). The histopathological and morphometric investigation of the effects of systemically administered boric acid on alveolar bone loss in ligature-induced periodontitis in diabetic rats. Acta Odontol Scand.

[44] Wu KL, Hsu C, Chan JY (2007). Impairment of the mitochondrial respiratory enzyme activity triggers sequential activation of apoptosis-inducing factor-dependent and caspase-dependent signaling pathways to induce apoptosis after spinal cord injury. J Neurochem.

[45] Li Q, Gao S, Kang Z, Zhang M, Zhao X, Zhai Y (2018). Rapamycin enhances mitophagy and attenuates apoptosis after spinal ischemia-reperfusion injury. Front Neurosci.

[46] Springer JE, Azbill RD, Knapp PE (1999). Activation of the caspase-3 apoptotic cascade in traumatic spinal cord injury. Nat Med.

[47] Yang C-H, Quan Z-X, Wang G-J, He T, Chen Z-Y, Li Q-C (2022). Elevated intraspinal pressure in traumatic spinal cord injury is a promising therapeutic target. Neural Regen Res.

[48] Koc ER, Gökce EC, Sönmez MA, Namuslu M, Gökce A, Bodur AS (2015). Borax partially prevents neurologic disability and oxidative stress in experimental spinal cord ischemia/reperfusion injury. J Stroke Cerebrovasc Dis.

[49] Cong L, Chen W (2016). Neuroprotective effect of ginsenoside Rd in spinal cord injury rats. Basic Clin Pharmacol Toxicol.

